# Diversity and molecular epidemiology of HIV-1 in Cape Town; 1984 to 2010

**DOI:** 10.1186/1742-4690-8-S2-P21

**Published:** 2011-10-03

**Authors:** Susan Engelbrecht, Eduan Wilkinson, Tulio de Oliveira

**Affiliations:** 1 Division of Medical Virology, Stellenbosch University, Tygerberg, 7505, South Africa; 2 Africa Centre for Health and Population Studies, Nelson R Mandela School of Medicine, University of KwaZulu-Natal, South Africa

## Background

HIV-1 is a major health problem in South Africa with more than 6 million people infected. One of the features of the virus is the extreme genetic diversity, resulting in an uneven distribution of the different viral subtypes. This diversity may also impact on diagnostic assays, antiretroviral treatment, prevention and vaccine development. We investigated the diversity and molecular epidemiology of HIV-1 in the CapeTown area between 1984 and 2010.

## Materials and methods

Patient samples were collected from the Cape Town area between 1984 and 2010. HIV-1 RNA was extracted, amplified by RT-PCR and sequenced. High resolution phylogenetic analysis was used to investigate the molecular epidemiology and introduction of HIV-1 into South Africa. Maximum Likelihood trees were inferred in PhyMLv3.0 with the best nucleotide substitution model for each dataset. Bayesian genealogies were inferred in BEAST v1.6.1 using different substitution models, a relaxed molecular clock and different coalescent priors. A Markov chain Monte Carlo (MCMC) chain was run for 10,000,000 generations with sampling every 10,000^th^ generation using super computer clusters. Results were visualized in Tracer v1 .5.

## Results

Phylogenetic analysis indicated two different epidemics in Cape Town: HIV-1 subtypes B and D in homosexual men and subtype C in the heterosexual population. Molecular clock and likelihood mapping analysis indicated that HIV-1 subtype C was introduced in South Africa, dating back to the 1960-1970s, much earlier than previously thought. More recent samples also indicated that the epidemic is evolving with the frequent detection of unique recombinant forms (URFs) which may be the precursors of emerging CRFs in the future.

## Conclusion

The detection of URFs may indicate a change in the dynamics of the predominant subtype C epidemic in South Africa. It is essential to study HIV-1 in order to improve our knowledge of these viruses and to obtain a better understanding of the viruses in circulation in South Africa.

